# Decomposition of gender disparities in abnormal sleep duration among Chinese older adults: evidence from a national survey

**DOI:** 10.3389/fpubh.2026.1903876

**Published:** 2026-10-28

**Authors:** Na Zhang, Mengqian Wu, Kaijun Han, Manlan He, Lu Chen

**Affiliations:** 1 Department of Nursing, Nanjing Drum Tower Hospital, Affiliated Hospital of Medical School, Nanjing University, Nanjing, China; 2 Department of Neurosurgery, Nanjing Drum Tower Hospital, Affiliated Hospital of Medical School, Nanjing University, Nanjing, China

**Keywords:** Chinese, decomposition analysis, gender, older adults, sleep duration

## Abstract

**Background:**

Sleep duration is an important component of healthy aging, yet gender differences in short and long sleep among older adults remain insufficiently understood. This study aimed to examine gender differences in sleep duration among Chinese older adults and quantify the contributions of socioeconomic, behavioral, functional, and health-related factors to these differences.

**Methods:**

Data were obtained from 9,590 adults aged ≥65 years who participated in the 2018 wave of the Chinese Longitudinal Healthy Longevity Survey. Sleep duration was self-reported and categorized as short (<6 h), normal (6–8 h), or long (>8 h). Multinomial logistic regression was used to identify factors associated with short and long sleep, with weighted analyses and Poisson regression performed as sensitivity analyses. Separate Fairlie decomposition analyses were conducted for short and long sleep to quantify the contributions of observed characteristics to gender differences.

**Results:**

Overall, 44.95% of participants had abnormal sleep duration, with a higher prevalence among women than men (46.87% vs. 42.66%, *p* < 0.001). Compared with men, women had a higher relative risk of short sleep (RRR = 1.399, 95% CI: 1.204–1.625) but a lower relative risk of long sleep (RRR = 0.856, 95% CI: 0.754–0.972). The association with short sleep remained significant in the weighted analysis (RRR = 1.530, 95% CI: 1.210–1.935), whereas the gender difference in long sleep was attenuated (RRR = 0.821, 95% CI: 0.661–1.021). For short sleep, observed characteristics explained 21.65% of the gender disparity. Education (22.75%), depressive symptoms (11.07%), IADL limitation (6.02%), and self-assessed health (4.82%) contributed positively to the disparity, whereas smoking status (−22.57%), drinking status (−12.45%), and exercise (−4.87%) partially offset it. The gender difference in long sleep was negligible, with substantial offsetting contributions across factors.

**Conclusion:**

Gender differences in sleep duration among Chinese older adults were primarily concentrated in short sleep, with women experiencing a higher risk. Socioeconomic, behavioral, psychological, and functional characteristics contributed to this disparity, although most of the short-sleep difference remained unexplained. Gender-sensitive interventions addressing educational disadvantage, depressive symptoms, functional limitations, and health behaviors may help reduce inequalities in sleep duration among older adults.

## 1 Introduction

Aging has become a challenge worldwide, especially in low-and middle-income countries. In 2022, China’s population aged 60 and over reached 280 million; By 2050, China will have 400 million people over the age of 65 (26.9% of the total population), which indicates that China will become one of the countries with the highest proportion of older people in the world (1). With advancing age, significant changes occur in sleep architecture, including a diminished ability to maintain sleep (increased awakenings and prolonged nighttime wakefulness), reduced total nighttime sleep duration, and a decline in deep sleep (slow-wave sleep) (2).

Abnormal sleep duration are a significant health issue affecting older adults, with their prevalence increasing with age (3). Among these, abnormal sleep duration (either too short or too long) is one of the common manifestations. A strong non-linear U-shaped or V-shaped association has been observed between sleep duration and various health conditions, including cardiovascular disease, cognitive decline, coronary heart disease, depression, falls, frailty, lung cancer, multiple sclerosis, osteoarthritis, and stroke (4, 5). Sleep duration in older adults is influenced by multiple factors, such as physical diseases and mental health conditions, as well as environmental, social, and lifestyle changes, among which gender is an important factor (6).

Gender differences have been reported in brain development and many clinical and psychosocial conditions. Sleep problems increase with age in both men and women (7). Although men and women sleep differently, women generally experience poorer quality and more chaotic sleep at all stages of life (8). At present, most of the studies on gender differences in sleep duration focus on gender differences in the relationship between sleep duration and cognition, depression, sarcopenia, visual impairment, hypertension, obesity, and mortality, but few studies focus on gender differences in sleep duration (9–12). A survey of healthy Korean older adults living in the community found significant gender differences in sleep changes, with women sleeping shorter, sleeping longer, and sleeping more efficiently over 6 years. Sleep patterns deteriorated in both groups, but more pronounced changes were observed in women (13). Given the close link between sleep patterns and sociodemographic factors, there is a need for region-specific studies.

Currently, empirical data on the prevalence and determinants of abnormal sleep duration among Chinese older adults remains scarce, with a notable paucity of research examining potential gender-specific variations. To address this gap, this study utilizes data from the Chinese Longitudinal Healthy Longevity Survey (CLHLS) to preliminarily identify factors associated with sleep duration. Furthermore, the Fairlie decomposition method was employed to quantify the relative contributions of these factors to the observed gender differences in sleep duration. The findings aim to offer an evidence-based foundation for developing targeted interventions designed to improve sleep duration among the older adults population in a gender-specific manner.

## 2 Methods

### 2.1 Study population

Our data are from the Chinese Longitudinal Healthy Longevity Survey (CLHLS). The database was previously described (14). The survey was conducted nationally between 1998 and 2018 with eight follow-up questionnaires containing very rich individual microdata on physical and psychological health, cognitive functioning, social participation, behavior, diet and nutrition, lifestyle habits, socioeconomic status, family structure, intergenerational relationships, older adults family care needs, care provision and costs of surviving older adults. The sampling framework of the database covers 23 provinces, autonomous regions, and municipalities directly under the central government. At the baseline survey, these regions accounted for approximately 85% of China’s total population, ensuring broad representativeness. A stratified sampling method by age was adopted, with deliberate oversampling of both the oldest-old (typically defined as those aged 80 and above) and male older adults participants. To maintain the stability of the age structure in the sample during long-term follow-up, the CLHLS research team implemented a sample replenishment strategy, replacing deceased or lost-to-follow-up participants with individuals of similar gender and age. These methodological measures collectively enhance the national representativeness of the sample for the older adults population in China. Data sources and data design reports for the project are available here^1^. This study followed the principles of the Declaration of Helsinki, and the informed consent of the participants was obtained before the investigation. The institutional review board approved the survey study of Peking University (No. IRB00001052-13074).

This study utilized data from the eighth follow-up survey (conducted in 2017–2018), which initially included 15,874 individuals aged 65 years or older. People with missing or incorrect responses for sleep duration, baseline characteristics, sociological characteristics, and personal lifestyle indicators were excluded, and a total of 9,590 cases were finally included in this study. The exclusion process is shown in Figure 1.

**Figure 1:**
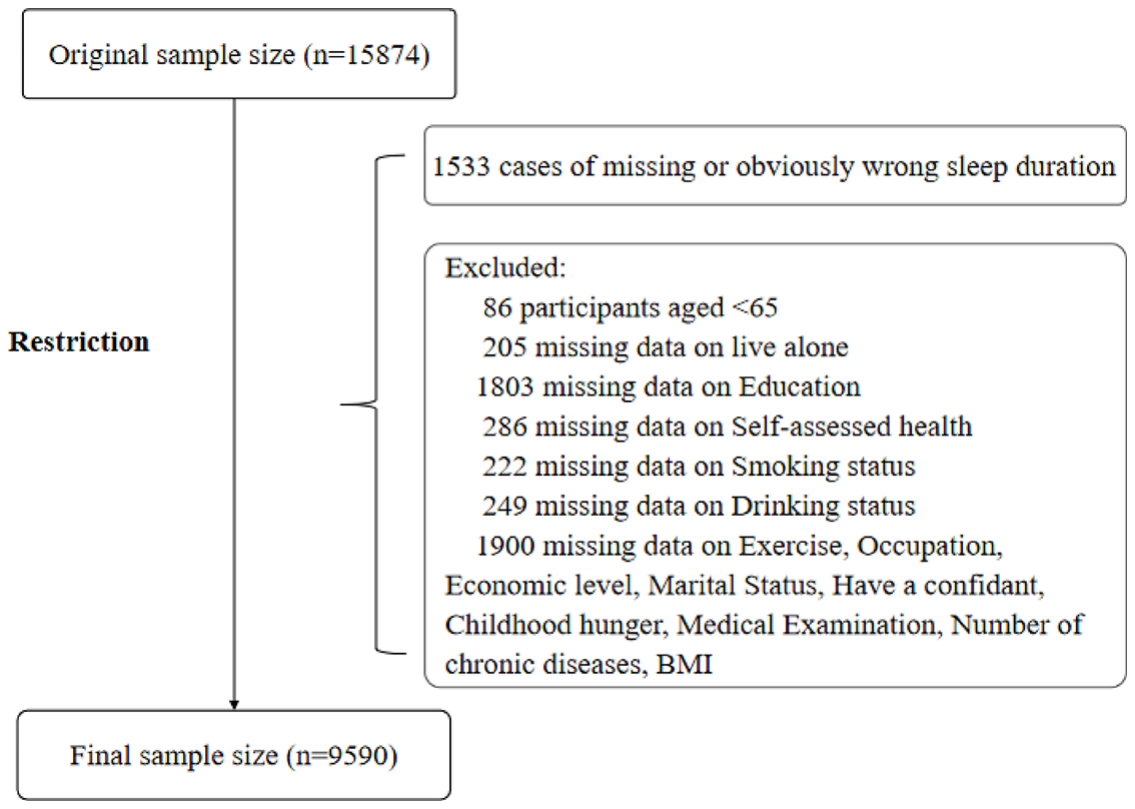
Flow chart of the selection process of participants in CLHLS.

### 2.2 Measures

#### 2.2.1 Self-reported sleep duration

Sleep duration was assessed using a single self-reported question: “On average, how many hours of sleep do you obtain within a 24-hour period?” to assess the amount of time participants slept. According to previous research findings, 6–8 h of sleep is beneficial among older adults (15), and a meta-analysis of 35 sleep studies (16). Study participants were divided into three main sleep duration: short (<6 h), normal (6–8 h), and long (>8 h) sleep duration.

##### 2.2.2 Independent variable

Baseline characteristics, chronic disease conditions, lifestyle, functional status, and social activities were considered independent variables in this study. All variables were assessed based on self-reports. Sociology-demographic characteristics included Residence (city or rural), sex (male or female), living alone (yes or no), education (No formal education, 1 to 6 years, seven or more), Occupation (Professional technician/doctor/teacher, Administration, General staff/ service worker/ worker, Freelancer, Farmers, Household work, Military, Unemployed, or other), Childhood hunger (yes or no), Economic level (Very rich, Wealthier, general, difficult, or very difficult), have a confidant (yes or no), Marital Status (Married and living with a spouse, Divorce, Widowed, Never married, or Married, but not living with a spouse), Medical Examination (yes or no). Chronic disease conditions included the number of chronic diseases (0, 1 ~ 2, or≥3) and BMI (<18.5, 18.5 ~ 23.9, 24.0 ~ 27.9, or ≥28.0). Functional status included self-assessed health (Excellent, Good, General, Poor, or Very poor), Social activities included exercise (yes or no), lifestyle included smoking status (Current, former, or never), and drinking status (Current, former, or never).

Depressive symptoms among older adults in the CLHLS were assessed using the 10-item Center for Epidemiological Studies Depression Scale (CESD-10). The scale contains 10 items: felt depressed, fearful, hopeful, unhappy, lonely, could not get going, bothered by little things, trouble concentrating, everything was an effort, and sleep was restless. Each item was scored from 0 (rarely or none of the time) to 3 (most or all of the time), with a higher score indicating more severe depressive symptoms. The CES-D10 has demonstrated good reliability and validity among older adults in China, with a Cronbach’s α of 0.815 (17). A cut-off point of 10 or higher on the total score for determining the presence of depressive symptoms (18).

Cognitive function of participants was measured using the Chinese version of the Mini-Mental State Examination (CMMSE) in all waves of the CLHLS. In the present study, we utilized the total CMMSE scores provided in the CLHLS dataset for secondary data analysis. The total possible score is 30, with a higher score indicating better cognitive function. Consistent with previous studies, a score below 24 was defined as cognitive impairment (19, 20). The Chinese version of the MMSE is a highly sensitive and specific tool for detecting dementia and is commonly used to evaluate cognitive function in the older adults (21).

The assessment of basic activities of daily living (ADL) was conducted using the scale originally developed by Katz and a health professional team (22). This scale evaluates six domains: bathing, dressing, indoor mobility, using the toilet, eating, and continence control. For each item, respondents selected from three options: completely independent, partially independent, or completely dependent.”A score of 1 was assigned if the participant reported needing assistance in any of the six activities; otherwise, a score of 0 was assigned (23).

The assessment of instrumental activities of daily living (IADL) covered eight domains: visiting neighbors alone, shopping alone, preparing meals alone, doing laundry alone, walking a distance of two kilometers, lifting a weight of five kilograms, squatting and standing up three times consecutively, and using public transportation alone. For each item, responses were categorized as able, able with some difficulty, or unable. A score of 1 was recorded if the respondent reported either “able with some difficulty” or “unable” in any of the eight activities; otherwise, a score of 0 was assigned (24, 25). IADL limitation may identify subtle reductions in independent functioning before basic self-care is affected, whereas ADL limitation indicates more advanced dependency. Assessing both measures helped avoid underestimating functional vulnerability and enabled their independent associations with short and long sleep duration, as well as their respective contributions to gender disparities, to be examined.

### 2.3 Statistical analysis

Descriptive statistics were used to analyze general information on baseline characteristics, sociological characteristics, individual lifestyles, and health status. A chi-square test was used to analyze the distribution characteristics of sleep duration among older adults of different genders. Sampling weights provided by the CLHLS were applied to estimate the weighted prevalence of abnormal, short, and long sleep duration in the overall sample and separately by gender, with corresponding 95% confidence intervals. Using normal sleep duration as the reference category, multinomial logistic regression models were fitted to separately identify factors associated with short and long sleep. The models were conducted in the overall sample and stratified by sex, and the results are presented as relative risk ratios (RRRs) with 95% confidence intervals. Because the Fairlie decomposition method requires a binary outcome, two separate decomposition analyses were conducted: short sleep versus normal sleep and long sleep versus normal sleep. For each comparison, the analysis was restricted to participants in the corresponding two sleep-duration categories. Pooled logistic regression coefficients for men and women were used to quantify the absolute and relative contributions of socioeconomic, behavioral, functional, and health-related factors to the gender disparities in short and long sleep. To reduce path dependence arising from variable ordering, the order of explanatory variables was randomized across 1,000 replications. Sampling weights were incorporated into the multinomial logistic regression and Fairlie decomposition analyses. The above statistical analysis was performed using Stata MP17.0 software. The level of statistical significance was defined as 0.05.

As the dependent variable is dichotomous, we used the Fairlie nonlinear decomposition method to decompose the sleep duration differences into the contributions of various factors. It is important to note that in the decomposition of nonlinear models, the results can be sensitive to the order in which variables are entered. To address this issue, we performed 100 iterations with random variable ordering and reported the averaged results (26). The formula is as follows:

$${\overset{-}{Y}}_{g}-{\overset{-}{Y}}_{s}=[\sum_{i=1}^{N^{g}}\frac{F(X_{i}^{g}\beta^{g})}{N^{g}}-\sum_{i=1}^{N^{s}}\frac{F(X_{i}^{s}\beta^{g})}{N^{s}}]+[\sum_{i=1}^{N^{s}}\frac{F(X_{i}^{s}\beta^{g})}{N^{g}}-\sum_{i=1}^{N^{s}}\frac{F(X_{i}^{s}\beta^{s})}{N^{s}}]$$

${\overset{-}{Y}}_{g}$ is the mean value of sleep duration for male, ${\overset{-}{Y}}_{s}$ is the mean value of sleep duration for female. $X_{i}^{g}$ explanatory variables for male, $X_{i}^{s}$ is a female older adults explanatory variable, β is the regression coefficient; $N^{g}$ is the sample size of male older adults, $N^{s}$ is the sample size of female older adults. The first half of the formula is the explainable part, i.e., the difference in sleep duration among older adults of different genders caused by the part that can be explained by other factors; The second half is the unexplained part, i.e., the differences caused by the regression coefficients of the length of sleep by gender.

## 3 Results

### 3.1 Baseline characteristics

A total of 9,590 participants were included in this study, of which 4,365 (45.52%) were men and 5,225 (54.48%) were women. We found that 22.91% of older adults had problems with sleep duration, with a higher prevalence of abnormal sleep duration in women than in men (*p* < 0.001). The results of the chi-square analysis showed that men and women differed in terms of place of residence, age, whether they lived alone, self-rated health, smoking status, drinking status, exercise, education, occupation, economic level, marital status, childhood starvation, BMI, depression, cognitive and functional limitations (see Supplementary Table S1).

### 3.2 Multinomial logistic regression results

Using normal sleep duration (6–8 h) as the reference category, women had a higher relative risk of short sleep than men (RRR = 1.399, 95% CI: 1.204–1.625; *P* < 0.001) but a lower relative risk of long sleep (RRR = 0.856, 95% CI: 0.754–0.972; *p* = 0.017). Short sleep was positively associated with living alone, poorer self-assessed health, lower economic status, depressive symptoms, IADL impairment, and a greater number of chronic diseases, whereas smoking status, exercise, higher BMI category, and higher educational level were inversely associated with short sleep (all *p* < 0.05). Long sleep was positively associated with rural residence, older age, occupation, lack of a medical examination, cognitive impairment, ADL impairment, and IADL impairment, whereas smoking status, depressive symptoms, and higher BMI category were inversely associated with long sleep (all *p* < 0.05) (Table 1).

**Table 1:** Multinomial logistic regression of factors associated with short and long sleep duration.

Variable	Short sleep vs. normal sleep			Long sleep vs. normal sleep		
	RRR	*p* value	95% CI	RRR	*p* value	95% CI
Gender	1.399	<0.001	1.204–1.625	0.856	0.017	0.754–0.972
Residence	1.011	0.896	0.861–1.187	1.271	0.001	1.105–1.463
Age group	1.075	0.166	0.970–1.190	1.313	<0.001	1.195–1.442
Living alone	1.197	0.026	1.022–1.402	0.897	0.150	0.774–1.040
Self-assessed health	1.282	<0.001	1.196–1.375	0.951	0.111	0.894–1.012
Smoking status	0.891	0.015	0.812–0.978	0.882	0.002	0.816–0.953
Drinking status	0.927	0.085	0.850–1.010	0.939	0.098	0.872–1.012
Exercise	0.793	<0.001	0.700–0.898	0.954	0.415	0.853–1.068
Occupation	1.031	0.179	0.986–1.079	1.060	0.003	1.020–1.101
Economic level	1.247	<0.001	1.136–1.369	1.002	0.972	0.921–1.089
Marital status	0.996	0.869	0.948–1.046	1.036	0.219	0.991–1.082
Childhood hunger	0.909	0.163	0.795–1.040	1.006	0.919	0.896–1.130
Medical examination	0.997	0.969	0.875–1.137	1.174	0.004	1.052–1.311
Having a confidant	0.803	0.175	0.585–1.103	0.895	0.488	0.654–1.225
Depression	2.274	<0.001	1.969–2.628	0.803	0.008	0.683–0.945
Cognitive function	1.008	0.918	0.862–1.180	1.414	<0.001	1.239–1.613
ADL	0.921	0.381	0.765–1.097	1.248	0.001	1.089–1.429
IADL	1.192	0.017	1.031–1.378	1.391	<0.001	1.217–1.590
BMI category	0.923	0.030	0.859–0.993	0.936	0.044	0.878–0.998
Educational level	0.807	<0.001	0.731–0.890	0.932	0.120	0.854–1.018
Number of chronic diseases	1.268	<0.001	1.159–1.387	1.006	0.822	0.931–1.087

Wald χ^2^(42) = 1,135.55; R^2^ = 0.0613; Normal sleep duration (6–8 h) was the base outcome. RRR, relative risk ratio; CI, confidence interval; ADL, activities of daily living; IADL, instrumental activities of daily living; BMI, body mass index.

The weighted multinomial logistic regression yielded broadly consistent results. After application of the CLHLS sampling weights, women remained more likely than men to report short sleep (RRR = 1.530, 95% CI: 1.210–1.935; *p* < 0.001), whereas the gender difference in long sleep was no longer statistically significant (RRR = 0.821, 95% CI: 0.661–1.021; *p* = 0.076) (Supplementary Table S3). The Poisson regression analyses with robust standard errors also supported associations of sleep duration with several sociodemographic, behavioral, and functional factors in both men and women (Supplementary Table S4, S5).

### 3.3 Decomposition analysis results

For short sleep, the observed gender difference was −0.0785, of which 21.65% was attributable to observed characteristics and 78.35% remained unexplained. Living alone (2.82%), self-assessed health (4.82%), education (22.75%), economic level (2.37%), depressive symptoms (11.07%), and IADL impairment (6.02%) made statistically significant positive contributions to the explained disparity. In contrast, smoking status (−22.57%), drinking status (−12.45%), and exercise (−4.87%) significantly offset the disparity (Table 2). For long sleep, the observed gender difference was only 0.0007. The absolute contributions of residence (*β* = −0.0011), age (*β* = −0.0120), medical examination (*β* = −0.0022), cognitive function (*β* = −0.0137), ADL (*β* = −0.0031), and IADL (*β* = −0.0119) opposed those of smoking status (*β* = 0.0259) and depressive symptoms (*β* = 0.0011), resulting in substantial offsetting effects. Because the observed long-sleep difference was extremely small, the corresponding percentage contributions were unstable and should be interpreted cautiously (Supplementary Table S2).

**Table 2:** Fairlie decomposition of short sleep duration disparity between male and female older adults.

Terms of decomposition	DS
Difference	−0.0785243
Explained	−0.01700240 (21.65%)
Non-explained	−0.06152193 (78.35%)
Explained	

Contribution to difference	*p*	*β*	Contribution (%)	95% CI
Residence	0.850	−0.0000968	0.12	−0.0010995, 0.0009059
Age	0.389	−0.0013371	1.70	−0.0043807, 0.0017064
Live alone	0.023	−0.0022114	2.82	−0.0041163, −0.0003065
Self-assessed health	<0.001	−0.0037878	4.82	−0.0051280, −0.0024476
Smoking status	0.007	0.0177248	−22.57	0.0047541, 0.0306955
Drinking status	0.043	0.0097795	−12.45	0.0003076, 0.0192514
Exercise	0.001	0.0038279	−4.87	0.0016069, 0.0060490
Education	<0.001	−0.0178681	22.75	−0.0276032, −0.0081330
Occupation	0.111	−0.0053629	6.83	−0.0119631, 0.0012374
Economic level	0.002	−0.0018632	2.37	−0.0030333, −0.0006930
Marital status	0.720	−0.0013480	1.72	−0.0087099, 0.0060139
Childhood hunger	0.132	−0.0005711	0.73	−0.0013150, 0.0001729
Medical examination	0.945	−0.0000350	0.04	−0.0010325, 0.0009624
Having a confidant	0.236	−0.0001211	0.15	−0.0003215, 0.0000794
Number of chronic diseases	0.969	−0.0000170	0.02	−0.0008661, 0.0008321
BMI	0.499	−0.0005658	0.72	−0.0022045, 0.0010728
Depression	<0.001	−0.0086934	11.07	−0.0104201, −0.0069666
Cognitive function	0.800	−0.0005053	0.64	−0.0044078, 0.0033973
ADL	0.276	0.0007981	−1.02	−0.0006377, 0.0022339
IADL	0.020	−0.0047235	6.02	−0.0086925, −0.0007546

*β* denotes the absolute contribution from the Fairlie decomposition. Contribution (%) = *β*/observed gender difference × 100. ADL, activities of daily living; IADL, instrumental activities of daily living; BMI, body mass index.

## 4 Discussion

This study explored the factors underlying gender differences in sleep duration among older adults in China, examining socio-demographic characteristics, lifestyle, and health status. Our analysis quantified the contribution of these factors to the disparity and confirmed that sleep duration significantly differs between older men and women.

In our study, 44.95% of the older adults had abnormal sleep duration, which is broadly consistent with previous reports suggesting that around 50% of Chinese older adults experience non-optimal sleep (27). The incidence of abnormal sleep duration in older adults women (46.87%) was higher than that in older adults men (44.95%), which was similar to the results of other scholars ‘studies on sleep duration in older adults China, indicating that there was a significant gender difference in sleep duration in older adults Chinese (28). A cross-sectional survey study of 675 older adults people in Sweden found that women had significantly shorter sleep duration than men (29); research in Nigeria also showed that women had more severe sleep problems compared to men (30). A study of 956 community-dwelling older adults aged 59–97 in Rotterdam found that whereas women subjectively reported poorer sleep, actigraphic measures objectively indicated more disrupted sleep in men (31). Our study places a greater emphasis on the Chinese older adults population, showed a 4.21% difference in the incidence of abnormal sleep duration between men and women, suggesting that activity in the suprachiasmatic nucleus, the primary pacemaker of the circadian clock, may also be affected by sex steroids (32). Women have a shorter circadian cycle than men (33), which may lead to a desynchrony between circadian time and sleep behavior. Suggests that women sleep later, leading to a lack of restorative sleep. Therefore, medical staff needs to pay more attention to the sleep duration of older women.

The multinomial logistic regression showed that short and long sleep had partly distinct correlates. Short sleep was associated with living alone, poorer self-assessed health, lower economic status, depressive symptoms, IADL limitation, and a greater number of chronic diseases. In contrast, long sleep was associated with rural residence, older age, occupation, lack of a medical examination, cognitive impairment, and limitations in ADL and IADL. These findings support the view that short and long sleep may represent different social and health-related processes rather than opposite ends of a single continuum (34–38). In contrast to other studies, we found that residence, physical examination, number of chronic diseases, and depression were the only risk factors for sleep duration in older women. Poor self-rated health status, occupation, and ADL dysfunction were the only risk factors for sleep duration in older men. Since older women living in rural areas have a lower socioeconomic status, are a vulnerable group, are less likely to have access to qualified living security and health care, and are more likely to have depressive symptoms that affect sleep (39) Consistent with one study, gender modulates sleep and IADL and ADL, with a stronger association in males (40). Poor self-rated health means they are less satisfied with their health and have difficulty participating in life with a positive attitude (41), which affects sleep.

The Fairlie decomposition provided additional insight into the sources of the gender disparity in short sleep. Observed characteristics explained 21.65% of the difference, while 78.35% remained unexplained. Education made the largest positive contribution among the measured factors (22.75%), followed by depressive symptoms (11.07%), IADL limitation (6.02%), self-assessed health (4.82%), living alone (2.82%), and economic level (2.37%). These results suggest that gender inequalities in educational opportunities, mental health, socioeconomic resources, and functional independence may contribute to women’s greater vulnerability to short sleep. The positive contribution of education is particularly relevant given the substantial educational disadvantage experienced by older Chinese women because of historical differences in access to schooling. Smoking status (−22.57%), drinking status (−12.45%), and exercise (−4.87%) made significant negative contributions to the short-sleep disparity. A negative contribution indicates that the gender distribution of the relevant factor offset, rather than widened, the observed difference. It should not be interpreted as evidence that smoking or drinking protects sleep. Older men had substantially higher rates of smoking and drinking than women, and these gender differences may have reduced the observed female–male disparity in short sleep. Both nicotine exposure and withdrawal can adversely affect sleep initiation and maintenance (42, 43). Consequently, the high smoking rate among males acts as a powerful negative factor that substantially lowers their average sleep duration. Without the influence of this smoking factor, the sleep disparity between males and females could be much larger.

In a novel approach, we employed decomposition analysis to examine sex differences in sleep duration. This study can lead to meaningful policy recommendations. First, we must pay attention to health education, encourage older people to develop good living habits, ensure adequate sleep time, actively exercise and participate in social activities. Secondly, increase the investment in geriatric care, encourage the community to actively participate in the geriatric health care program, focus on preventing and improving cognitive function, depressive symptoms, ADL, and IADL, and improve the health status of older people. Third, the government should pay more attention to disadvantaged groups, especially older women living in rural areas with low education levels and depression, and formulate targeted relief and assistance programs.

Our study has several limitations. First, sleep duration was based on self-report, which is subject to recall bias and may not precisely reflect objectively measured sleep. Second, although we adjusted for a broad range of socio-demographic, behavioral, functional, and health-related characteristics, several important sleep-related variables were unavailable or not included, such as sleep medication use, diagnosed sleep disorders, pain, nocturia, menopausal history, caregiving burden, and daytime napping. Some of these factors may differ systematically between older men and women and may also be independently associated with sleep duration, resulting in residual confounding. Consequently, the observed gender disparity and the estimated contributions of individual factors in the Fairlie decomposition may have been either overestimated or underestimated. In particular, part of the contribution attributed to measured factors, or included in the unexplained component, may reflect the effects of these unmeasured characteristics. Third, this study is cross-sectional and cannot verify the causal relationship between variables. Further research can be carried out through longitudinal investigation. Last, participants with extensive missing data were excluded, which may introduce selection bias.

## 5 Conclusion

The findings indicate that gender disparities in sleep duration among Chinese older adults were primarily evident in short sleep, with women having a higher risk than men. The measured characteristics explained 21.65% of the gender disparity in short sleep. Educational attainment, depressive symptoms, self-assessed health, and IADL limitation contributed positively to this disparity, whereas differences in smoking, drinking, and exercise partially offset it. In contrast, the gender difference in long sleep was small and was not statistically significant in the weighted analysis. These findings suggest that socioeconomic disadvantage, psychological health, functional limitations, and health behaviors are associated with gender disparities in short sleep. Gender-sensitive assessment and support targeting vulnerable older adults may help address inequalities in sleep duration, although longitudinal studies are needed to confirm these associations and their temporal relationships.

## Data Availability

The data analyzed in this study were obtained from the 2018 wave of the Chinese Longitudinal Healthy Longevity Survey (CLHLS) and are available through the Peking University Open Research Data Platform (https://opendata.pku.edu.cn/dataverse/CHADS). Access is subject to the platform’s data access requirements.

## Footnotes

1
Chinese Longitudinal Healthy Longevity and Happy Family Study (CLHLS-HF) (pku.edu.cn)
