# Supplementary material for: Decomposition of gender disparities in abnormal sleep duration among Chinese older adults: evidence from a national survey
Source: Front Public Health. 2026 Oct 28;14:1903876. doi: 10.3389/fpubh.2026.1903876 (PMC13751388; doi:10.3389/fpubh.2026.1903876)
Supplement: Supplementary file 1 [file Supplementary_file_1.pdf]

Supplementary Files

Table S1. Distribution of the variables in male and female respondents.

Variable	Total [n (%)]	Male [n (%)]	Female [n (%)]	χ^2	p
Sleep duration				17.06	< 0.001
Abnormal	4311(44.95)	1862(42.66)	2449(46.87)		
Normal	5279(55.05)	2503(57.34)	2776(53.13)		
Residence				10.65	0.001
City	2367(24.68)	1146(26.25)	1221(23.37)		
Rural	7223(75.32)	3219(73.75)	4004(76.63)		
Age				68.27	< 0.001
≤74	2565(26.75)	1274(29.19)	1291(24.71)		
75-89	3734(38.94)	1781(40.80)	1953(37.38)		
≥90	3291(34.32)	1310(30.01)	1981(37.91)		
Live alone				56.04	< 0.001
No	8026(83.69)	3788(86.78)	4238(81.11)		
Yes	1564(16.31)	577(13.22)	987(18.89)		
Self-assessed health				16.65	0.002
Very good	1177(12.27)	576(13.20)	601(11.50)		
Good	3386(35.31)	1591(36.45)	1795(34.35)		
General	3711(38.70)	1642(37.62)	2069(39.60)		
Poor	1217(12.69)	513(11.75)	704(13.47)		
Very poor	99(1.03)	43(0.99)	56(1.07)		
Smoking status				2900.3	< 0.001
Current	1528(15.93)	1308(29.97)	220(4.21)		
former	1482(15.45)	1279(29.30)	203(3.89)		
never	6580(68.61)	1778(40.73)	4802(91.90)		
Drinking status				1600.3	< 0.001
Current	1486(15.50)	1172(26.85)	314(6.01)		
former	1152(12.01)	896(20.53)	256(4.90)		
never	6952(72.49)	2297(52.62)	4655(89.09)		
Exercise					< 0.001
Yes	3350(34.93)	1764(40.41)	1586(30.35)		

No	6240(65.07)	2601(59.59)	3639(69.65)		
Education				1.4e+03	< 0.001
No formal education	4311(44.95)	1051(24.08)	3260(62.39)		
1 to 6	3285(34.25)	1954(44.77)	1331(25.47)		
7 or more	1994(20.79)	1360(31.16)	634(12.13)		
Occupation				752.23	< 0.001
Professional technician/ doctor/teacher	705(7.35)	461(10.56)	244(4.67)		
Administration	418(4.36)	325(7.45)	93(1.78)		
General staff/ service worker/ worker	1462(15.25)	757(17.34)	705(13.49)		
Freelancer	177(1.85)	105(2.41)	72(1.38)		
Farmers	5867(61.18)	2422(55.49)	3445(65.93)		
Household work	582(6.07)	80(1.83)	502(9.61)		
Military	87(0.91)	74(1.70)	13(0.25)		
Unemployed	121(1.26)	18(0.41)	103(1.97)		
Other	171(1.78)	123(2.82)	48(0.92)		
Economic level				45.58	< 0.001
Very rich	273(2.85)	160(3.67)	113(2.16)		
Wealthier	1651(17.22)	839(19.22)	812(15.54)		
general	6731(70.19)	2956(67.7)	3775(72.25)		
More difficult	817(8.52)	358(8.20)	459(8.78)		
Very difficult	118(1.23)	52(1.19)	66(1.26)		
Marital Status				895.99	< 0.001
Married and living with spouse	4289(44.72)	2607(59.73)	1682(32.19)		
Married, but not living with spouse	169(1.76)	110(2.52)	59(1.13)		
Divorce	28(0.29)	18(0.41)	10(0.19)		
Widowed	5029(52.44)	1567(35.90)	3462(66.26)		
Never married	75(0.78)	63(1.44)	12(0.23)		
Childhood hunger				10.81	< 0.001

Yes	6834(71.26)	3038(69.60)	3796(72.65)		
No	2756(28.74)	1327(30.40)	1429(27.35)		
Medical Examination				37.35	< 0.001
Yes	6808(70.99)	3234(74.09)	3574(68.40)		
No	2782(29.01)	1131(25.91)	1651(31.60)		
Have a confidant				1.807	0.179
No	263(2.74)	109(2.5)	154(2.95)		
Yes	9327(97.26)	4256(97.50)	5071(97.05)		
Number of chronic diseases				0.238	0.887
0	2545(26.54)	1161(26.60)	1384(26.49)		
1~2	4897(51.06)	2218(50.81)	2679(51.27)		
≥3	2148(22.40)	986(22.59)	1162(22.24)		
BMI				69.26	< 0.001
< 18.5	1455(15.17)	538(12.33)	917(17.55)		
18.5~23.9	4956(51.68)	2343(53.68)	2613(50.01)		
24.0~27.9	2370(24.71)	1159(26.55)	1211(23.18)		
≥28.0	809(8.44)	325(7.45)	484(9.26)		
Depression				36.56	< 0.001
No	8162(85.11)	3820(87.51)	4342(83.10)		
Yes	1428(14.89)	545(12.49)	883(16.9)		
Cognitive function				343.34	< 0.001
Normal	7270(75.81)	3696(84.67)	3574(68.40)		
Cognitive impairment	2320(24.19)	669(15.33)	1651(31.60)		
ADL				52.27	< 0.001
No	7693(80.22)	3642(83.44)	4051(77.53)		
Yes	1897(19.78)	723(16.56)	1174(22.47)		
IADL				299.54	< 0.001
No	3656(38.12)	2074(47.51)	1582(30.28)		
Yes	5934(61.88)	2291(52.49)	3643(69.72)		

Table S2. Fairlie decomposition of long sleep duration disparity between male and female older adults

Terms of decomposition			DS	
Difference			0.0007166	
Explained			-0.01919814 (-2679.06%)	
Non-explained			0.01991474 (2779.06%)	
Explained				
Contribution to difference	*p*	*β*	Contribution (%)	95% CI
Residence	0.015	-0.0011327	-158.07	-0.0020453, -0.0002201
Age	<0.001	-0.0119951	-1673.89	-0.0162228, -0.0077622
Live alone	0.166	0.0010554	147.28	-0.0004372, 0.0025480
Self-assessed health	0.056	0.0006853	95.63	-0.0000165, 0.0013871
Smoking status	<0.001	0.0258500	3607.31	0.0118498, 0.0398503
Drinking status	0.054	0.0089259	1245.59	-0.0001682, 0.0180201
Exercise	0.484	0.0009224	128.72	-0.0016581, 0.0035030
Education	0.384	-0.0050832	-709.35	-0.0165299, 0.0063636
Occupation	0.538	-0.0023618	-329.58	-0.0098731, 0.0051496
Economic level	0.776	0.0002193	30.60	-0.0012942, 0.0017329
Marital status	0.189	-0.0058254	-812.92	-0.0145187, 0.0028680
Childhood hunger	0.711	0.0001204	16.80	-0.0005168, 0.0007577
Medical examination	0.011	-0.0022426	-312.95	-0.0039725, -0.0005127
Having a confidant	0.699	-0.0000517	-7.21	-0.0003138, 0.0002104
Number of chronic diseases	0.931	-0.0000229	-3.20	-0.0005411, 0.0004954
BMI	0.518	-0.0006365	-88.82	-0.0025645, 0.0012915
Depression	0.005	0.0010932	152.55	0.0003349, 0.0018515
Cognitive function	<0.001	-0.0136817	-1909.25	-0.0191274, -0.0082361
ADL	0.011	-0.0031203	-435.43	-0.0055245, -0.0007162

IADL	<0.001	-0.0119010	-1660.76	-0.0168396, -0.0069625

Note: β denotes the absolute contribution from the Fairlie decomposition. Contribution (%) = β / observed gender difference × 100. Because the observed difference in long sleep prevalence was very small (0.0007166), percentage contributions are large and reflect substantial offsetting positive and negative contributions. ADL, activities of daily living; IADL, instrumental activities of daily living; BMI, body mass index.

Table S3. Weighted multinomial logistic regression of factors associated with short and long sleep duration

**Variable**	**Short sleep vs normal sleep**			**Long sleep vs normal sleep**		
	**RRR**	**P value**	**95% CI**	**RRR**	**P value**	**95% CI**
Gender	1.530	<0.001	1.210–1.935	0.821	0.076	0.661–1.021
Residence	0.902	0.415	0.705–1.156	1.564	<0.001	1.236–1.978
Age group	1.161	0.057	0.996–1.353	1.247	0.003	1.076–1.445
Living alone	1.163	0.245	0.902–1.501	0.954	0.714	0.743–1.225
Self-assessed health	1.395	<0.001	1.251–1.556	1.061	0.223	0.964–1.168
Smoking status	0.905	0.164	0.786–1.042	0.962	0.531	0.852–1.086
Drinking status	0.818	0.002	0.720–0.930	0.928	0.214	0.825–1.044
Exercise	0.882	0.162	0.740–1.052	0.918	0.329	0.773–1.090
Occupation	1.080	0.035	1.005–1.159	1.087	0.006	1.025–1.153
Economic level	1.244	0.004	1.071–1.446	1.038	0.583	0.909–1.184
Marital status	0.989	0.752	0.921–1.061	1.018	0.614	0.950–1.090
Childhood hunger	0.905	0.320	0.742–1.102	1.140	0.169	0.946–1.374
Medical examination	0.969	0.765	0.786–1.194	0.994	0.951	0.816–1.210
Having a confidant	0.736	0.208	0.456–1.186	0.909	0.696	0.563–1.467
Depression	2.267	<0.001	1.820–2.825	0.598	<0.001	0.453–0.790
Cognitive function	1.049	0.744	0.788–1.396	1.438	0.007	1.105–1.870
ADL	1.044	0.798	0.751–1.452	1.501	0.005	1.133–1.987
IADL	1.142	0.181	0.940–1.387	1.286	0.010	1.063–1.556

BMI category	0.911	0.082	0.820–1.012	0.917	0.110	0.825–1.020
Educational level	0.775	<0.001	0.674–0.891	0.818	0.004	0.714–0.938
Number of chronic diseases	1.197	0.009	1.046–1.370	1.010	0.874	0.891–1.145

Note: Normal sleep duration (6 – 8 h) was the base outcome. RRR, relative risk ratio; CI, confidence interval; ADL, activities of daily living; IADL, instrumental activities of daily living; BMI, body mass index. Sampling weights and robust standard errors were used.

Table S4. Poisson Regression of Factors Associated with Sleep Duration in Older men

Variable	Coefficient	Robust SE	*p*	95%*CI*	
Age	0.066	0.014	＜0.001	0.038	0.094
Residence	0.030	0.021	0.169	-0.013	0.072
Live alone	0.002	0.024	0.931	-0.045	0.050
Self-assessed health	0.010	0.010	0.308	-0.009	0.029
Smoking status	-0.040	0.010	＜0.001	-0.059	-0.021
Drinking status	0.002	0.009	0.849	-0.017	0.020
Exercise	-0.012	0.016	0.480	-0.044	0.020
Education	-0.006	0.002	0.013	-0.010	-0.001
Occupation	0.014	0.005	0.012	0.003	0.024
Economic level	-0.001	0.012	0.945	-0.025	0.023
Marital Status	0.000	0.007	0.942	-0.012	0.013
Childhood hunger	0.013	0.018	0.447	-0.021	0.048
Medical Examination	0.014	0.017	0.418	-0.020	0.048
Have a confidant	0.024	0.046	0.607	-0.066	0.113
Number of chronic diseases	0.000	0.005	0.952	-0.009	0.010
BMI	-0.019	0.010	0.059	-0.039	0.001
Depression	0.071	0.022	0.001	0.028	0.113
Cognitive function	0.060	0.020	0.003	0.021	0.100
ADL	-0.042	0.022	0.060	-0.086	0.002
IADL	0.066	0.020	0.001	0.028	0.105

Note: SE-Robust Standard Error, 95% CI-Confidence Intervals

Table S5. Poisson Regression of Factors Associated with Sleep Duration in Older women

Variable	Coefficient	Robust SE	*p*	95%*CI*	
Age	0.035	0.013	0.006	0.010	0.060
Residence	0.043	0.020	0.029	0.004	0.082
Live alone	-0.031	0.018	0.087	-0.066	0.004
Self-assessed health	-0.011	0.008	0.177	-0.027	0.005
Smoking status	0.000	0.015	0.995	-0.029	0.029
Drinking status	-0.047	0.013	＜0.001	-0.073	-0.022
Exercise	-0.017	0.016	0.260	-0.048	0.013
Education	-0.006	0.003	0.023	-0.011	-0.001
Occupation	0.004	0.006	0.472	-0.008	0.017
Economic level	0.013	0.011	0.267	-0.010	0.035
Marital Status	0.013	0.006	0.028	0.001	0.025
Childhood hunger	-0.011	0.016	0.492	-0.043	0.021
Medical Examination	0.046	0.015	0.002	0.017	0.075
Have a confidant	-0.050	0.037	0.182	-0.122	0.023
Number of chronic diseases	0.004	0.004	0.320	-0.004	0.012
BMI	-0.011	0.008	0.194	-0.027	0.006
Depression	0.030	0.018	0.094	-0.005	0.066
Cognitive function	0.068	0.018	＜0.001	0.033	0.103
ADL	0.020	0.017	0.241	-0.013	0.053
IADL	0.055	0.016	0.001	0.023	0.088

Note: SE-Robust Standard Error, 95% CI-Confidence Intervals
